# Vaccination coverage and its determinants among migrant children in Guangdong, China

**DOI:** 10.1186/1471-2458-14-203

**Published:** 2014-02-26

**Authors:** Ke Han, Huizhen Zheng, Zhixiong Huang, Quan Qiu, Hong Zeng, Banghua Chen, Jianxiong Xu

**Affiliations:** 1 Department of Pathogen Biology, School of Public Health and Tropical Medicine, South Medical University, Guangzhou, China; 2 Department of Immunization Program, Guangdong Center for Disease Control and Prevention, Guangzhou, Guangdong, China; 3 Department of Immunization Program, Nan Hai Center for Disease Control and Prevention, Nanhai, Foshan, Guangdong, China; 4 Department of Acute Infectious Disease, Wuhan Center for Disease Control and Prevention, Wuhan, Hubei, China; 5 Department of Immunization Program, Guangzhou Center for Disease Control and Prevention, Guangzhou, Guangdong, China

**Keywords:** Up-to-date, Age-appropriate immunization, Immunization coverage, Risk factor, Migrant children

## Abstract

**Background:**

Guangdong province attracted more than 31 million migrants in 2010. But few studies were performed to estimate the complete and age-appropriate immunization coverage and determine risk factors of migrant children.

**Methods:**

1610 migrant children aged 12–59 months from 70 villages were interviewed in Guangdong. Demographic characteristics, primary caregiver’s knowledge and attitude toward immunization, and child’s immunization history were obtained. UTD and age-appropriate immunization rates for the following five vaccines and the overall series (1:3:3:3:1 immunization series) were assessed: one dose of BCG, three doses of DTP, OPV and HepB, one dose of MCV. Risk factors for not being UTD for the 1:3:3:3:1 immunization series were explored.

**Results:**

For each antigen, the UTD immunization rate was above 71%, but the age-appropriate immunization rates for BCG, HepB, OPV, DPT and MCV were only 47.8%, 45.1%, 47.1%, 46.8% and 37.2%, respectively. The 1st dose was most likely to be delayed within them. For the 1:3:3:3:1 immunization series, the UTD immunization rate and age-appropriate immunization rate were 64.9% and 12.4% respectively. Several factors as below were significantly associated with UTD immunization. The primary caregiver’s determinants were their occupation, knowledge and attitude toward immunization. The child’s determinants were sex, Hukou, birth place, residential buildings and family income.

**Conclusions:**

Alarmingly low immunization coverage of migrant children should be closely monitored by NIISS. Primary caregiver and child’s determinants should be considered when taking measures. Strategies to strengthen active out-reach activities and health education for primary caregivers needed to be developed to improve their immunization coverage.

## Background

China had achieved remarkable economic growth through the development of market economy, but regional income imbalance became larger in recent years. In China, eastern regions’ annual incomes per capita for urban and rural households were much higher than those in western regions. These regional differences had become the important driving force of the country’s largest tide of internal migration. According to China’s sixth Population Census in 2010, there were more than 221 million internal migrants and which was 117% higher than the result of fifth Population Census in 2000 [1]. As a developed province in southeast areas of China, Guangdong alone had attracted more than 21 million migrants in 2000. In 2010, the number of migrants in Guangdong increased to 31 million and the amplification was the highest in China [1]. “Migrants” referred to people living in places other than their hometowns and possessing no local household registration card (Hukou) of their current living address; whereas “registered people” were those living in their hometowns and owning the local Hukou [2].

Vaccination has been regarded as one of the most important achievements of public health [3]. The migration of children from one region to another had been found to be associated with low vaccination coverage [4]. More than 90% of registered children aged 1–3 years completed the primary doses of Bacille Calmette Guerin (BCG), diphtheria, tetanus and pertussis combined vaccine (DTP), hepatitis B vaccine (HepB), oral poliomyelitis vaccine (OPV), and measles-containing vaccine (MCV) [5]. However, less than 70% migrant children of 1–3 years completed those immunizations [6].

Vaccination coverage was hampered by difficulty in accessing medical care, costs, complex transport and storage requirements, and by users characteristics, such as low education, parental knowledge, attitude and family poverty [7-11]. In some developed countries, risk factors for low vaccination of children at low socioeconomic level were explored and effective strategies were implemented [12,13]. In Guangdong, the immunization system differed from that in developed countries: all children were vaccinated in public clinics which did not provide medical services, and all Expanded Program of Immunization (EPI) vaccines were purchased by central government and provided to registered and migrant children free of charge. Migrant children have been covered in Guangdong’s EPI and thus have access to the same free vaccine and vaccination services as those registered children. Thus risk factors for low vaccination of migrant children in Guangdong may differ from that in developed countries. Furthermore, evaluation of age-appropriate vaccination coverage of migrants was very limited and risk factors from the perspective of immunization provision were rarely reported if not absence.

Due to much lower vaccination coverage of migrant children than that of registered children (65% vs 96%) [6] interventions specially targeted at migrant children in these areas were greatly needed. In 2010, a project financed by United Nations International Children’s Emergency Fund (UNICEF) was launched to explore effective strategies to improve immunization coverage of migrant children in Guangdong. Our research was conducted to estimate the actual complete and age-appropriate immunization coverage of migrant children in project areas and determine the risk factors of up-to-date(UTD) immunization.

## Methods

### Study population and vaccination schedule

The study population in our survey was the migrant children aged 12–59 months who had lived in the surveyed counties continuously for 3 months or more at the time of interview. Children were excluded if they were born in other provinces and had been living in the surveyed county for less than 3 months. Four vaccines of BCG, DTP, OPV and MCV have been included in EPI in China since 1978. In 1992, HepB was introduced into the routine immunization schedule but provided free of charge since 2002. Since then, there were no changes occurred in the immunization schedule for primary doses of the five vaccines. National recommended ages for primary doses of these five vaccines were showed in Table 1. In 2005, the government made a decision that all these vaccines were purchased by the government and provided to all children (including migrant children) free of charge, without any extra service fee. Guangdong province follows the national immunization schedule.

**Table 1:** Recommended age, acceptable age for the 1st dose and acceptable intervals between doses of the studied vaccines: Guangdong, China, 2011

	**Recommended age for routine immunization (month)**	**Acceptable age for the 1st dose, month (days)**^**a**^	**Acceptable interval between doses, month (days)**^**a**^
BCG	birth	birth (0–1)	-
HepB			
1	birth	birth (0–1)	-
2	1	-	1 (28–61)
3	6	-	59–183
OPV			
1	2	2 (59–91)	-
2	3	-	28–61
3	4	-	28–61
DPT			
1	3	3 (89–122)	-
2	4	-	28–61
3	5	-	28–61
MCV	8	8 (242–274)	-

^a^The minimum acceptable age for the 1st dose and the minimum acceptable interval between doses were approved by China’s MOH^17^. The maximum acceptable age for the 1st dose and the maximum acceptable interval between doses were determined by the recommended age for routine administration.

### Immunization coverage survey

The sampling method was based on the WHO-advocated cluster sampling technique [14]. Two districts of Nanhai in Foshan and Tianhe in Guangzhou where migrant people lived densely were surveyed. All of 429 villages within 29 towns and townships served as clusters. Primary immunization coverage for studied five vaccines of migrant children aged 12–59 months was assumed to be 75% [9] and the desired precision was ±3%. We assumed a design effect of two and obtained required number of children per cluster for variable numbers of clusters through the table recommended by WHO manual. Twenty-three children per cluster for 70 clusters were finally decided as our sample size.

We used a two-stage random sampling method. Initially, the list of 429 villages in the 29 towns and townships was obtained. Seventy clusters were selected using probability-proportionate-to-size (PPS) of migrant children aged 12–59 months in Guangdong’s immunization activity for OPV in 2010. Interviewers got the list of migrant household from local community authorities in each selected village and randomly selected one (using random numbers) as the first family of an eligible child to be interviewed. Then interviewers visited each subsequent household located to the right of the previous one until 23 eligible children were interviewed. Only one child per family was selected to avoid clustering. When two or more eligible children were in the same household, the youngest child was selected based on WHO’s manual [14]. A total of 1530 eligible children were surveyed.

One interview team was comprised of two interviewers and a local guide. EPI staffs of district center for disease control and prevention (CDC) and township were trained as interviewers. Local guides were selected from community authorities of surveyed villages to familiarize interviewers with local circumstances and introduce interviewers to the surveyed household. Provincial CDC staffs supervised the quality of investigation. Standard face-to-face interviews were conducted with primary caregivers, who were mothers (72.6%) or fathers (16.8%) in mothers’ absence or other family members (10.6%) in parental absence.

The interviewer used a standard questionnaire Additional file 1, including demographic characteristics of the surveyed child and the primary caregiver; the child’s migrant information; the primary caregiver’s knowledge and attitude toward immunization. Caregivers were questioned as to their knowledge about immunization, including what vaccines were given to children for free by government and what diseases were prevented by these vaccines: responses were scored by the number correct. The attitude scores were obtained based on their awareness toward immunization safety and frequency of Adverse Events Following Immunization (AEFI). The high scores indicated the high level of knowledge and attitude. Only written vaccination history (the immunization certificate kept by caregivers or the immunization card kept by vaccination clinics) was accepted as the proof of immunization [15], because caregivers’ recall of their child’s immunization history was unreliable.

### Data analysis

The coverage of vaccination status was estimated for each dose, each vaccine series and the 1:3:3:3:1 immunization series (one dose BCG; three doses of HepB; three doses of DTP; three doses of OPV and one dose of MCV). We classified the outcome into three categories: invalid immunization, age-appropriate immunization, and delayed immunization.

UTD immunization rate was defined as the percentage of children completing required doses of vaccines regardless of immunization age of each dose. We used immunization date and birth date to calculate immunization age. Acceptable ages for the 1st dose and intervals between doses were converted to days. Each range of acceptable ages or intervals began at the smallest number of days and end at the greatest number of days composing the given number of months. For example, the acceptable age for the 1st DTP was 3 months, so the dose received at 89–122 days was age-appropriate (Table 1). The 1st dose received before the minimum acceptable age of the 1st dose and the following doses received before the minimum acceptable intervals between doses were defined age-invalid and interval-invalid. Accordingly, the 1st dose received after maximum acceptable age and the following doses received after maximum acceptable intervals were considered age-delayed and interval-delayed, respectively. Vaccine series were defined age-appropriate if the 1st dose and the following doses were received at the acceptable age and intervals, respectively.

Logistic regression analysis was used to analyze the variables (socio demographic variables, knowledge about vaccine and disease, and attitude toward immunization safety) independently associated with UTD immunization rate (p < 0.05). The dependent variable was dichotomized as 'UTD /not UTD’ for the five vaccine series. All the analyses above utilized SPSS statistical software, version 17.0.

### Ethical considerations

The survey was approved by the Institutional Ethics Committee of Guangdong Center for Disease Control and Prevention. The Written Informed consent was obtained from the participants (child’s parents or guardians) prior to participation in the survey, and data collection was conducted confidentially.

## Results

Among 1530 children in our survey, 49 had neither certificates nor cards and were regarded as unvaccinated. Among the rest 1481 children, 1475 had certificates, 1438 had cards, and 1437 had both. Among those 1437 children, 18 had different vaccination records between their certificates and cards. Further comparison of these 18 pairs of written immunization history showed that 11 certificates recorded more vaccine doses than the paired cards, and the other seven pairs recorded same vaccine doses but different vaccination dates. Based on ministry of health (MOH)’s guideline [16], the child’s immunization history was assessed from the certificate kept by caregivers, and if no certificate was available, the immunization card kept by clinics was used. The dose was considered unreceived if its vaccination date was missing on the written history.

### Coverage of immunization status for each primary dose, each vaccine series and the 1:3:3:3:1 immunization series

Table 2 summarized the UTD and the age-appropriate immunization rate of each primary dose of the studied five vaccines. For HepB, OPV, and DPT series, the coverage decreased with doses. The coverage gap between the 1^st^ dose and 3rd dose ranged from 2.5% for OPV series to 5.2% for HepB series. For each antigen, the UTD immunization coverage rate was above 71%, but the age-appropriate immunization rates were much lower as 47.8%, 45.1%, 47.1%, 46.8% and 37.2% for BCG, HepB, OPV, DPT and MCV, respectively. For the1:3:3:3:1 immunization series, the UTD immunization rate was 64.9%, but the age-appropriate immunization rate was only 12.9%. Within BCG, HepB, OPV, DPT and MCV series, the 1st dose suffered the highest delayed rates ranged from 13.1% to 32.8%. Within HepB, OPV and DPT series, the 2nd and 3rd dose suffered the interval-delayed rates from 5.9% to 14.3%. For all studied doses, delayed immunization accounted for most of not age-appropriate immunization.

**Table 2:** Coverage of immunization status for each primary dose and the 1:3:3:3:1 immunization series: Guangdong, China, 2011

	**Coverage (%)**	**Invalid (%)**		**Age-appropriate (%)**	**Delayed (%)**	
		**Age-invalid**	**Interval-invalid**		**Age-delayed**	**Interval-delayed**
BCG	76.3	.0		47.8	28.5	
HepB1	76.7	.0		63.6	13.1	
HepB2	75.8		1.0	68.8		5.9
HepB3	71.5		1.2	55.9		14.3
OPV1	77.6	1.9		58.8	16.9	
OPV2	76.6		1.3	67.3		8.0
OPV3	75.1		2.0	64.2		8.9
DPT1	76.6	1.4		60.4	14.8	
DPT2	75.3		1.0	66.7		7.5
DPT3	73.7		0.8	60.5		12.4
MCV1	71.8	1.8		37.2	32.8	
The 1: 3:3:3:1 immunization series^a^	64.9	1.9		12.4	50.6	

^a^The1:3:3:3:1 immunization series: one dose of BCG, three doses of hepatitis B vaccine, three doses of oral poliovirus vaccine, three doses of diphtheria and tetanus toxoids and pertussis vaccine, and one dose of measles-containing vaccine.

### Influence factors for being UTD for the1:3:3:3:1 immunization series

Results of logistic regression analyses (Table 3) showed that compared with workers, the primary caregiver’s occupation of commercial staff (odds ratio (OR) =1.87, 95%CI 1.27, 2.76), professionals (OR = 2.00, 95%CI 1.04, 3.83), private owners (OR = 2.23, 95%CI 1.47, 3.37), housewives (OR = 1.67, 95%CI 1.19, 2.35), increased the odds of being UTD for the 1:3:3:3:1 immunization series. The primary caregiver’s knowledge about vaccine and disease increased the child’s odds of being UTD for the 1:3:3:3:1 immunization series. Compared with primary caregivers whose knowledge scores <9, people with 10–19 scores (OR = 2.31; 95%CI 1.83, 2.91) and 20–34 scores (OR = 3.33; 95%CI 2.38, 4.67) were more likely to receive UTD immunization. The child whose primary caregivers had 8–13 attitude scores toward immunization safety was more likely to be UTD for the series (OR =1.75; 95%CI 1.40, 2.19). Family income more than 3000 Yuan/person/year increased child’s odds ratio of being UTD. Girls were less likely received UTD immunization compared with boys (OR =0.76; 95%CI 0.62, 0.93). Compared with children owning Hukou out of Guangdong, children owning Guangdong Hukou were more likely to receive UTD immunization (OR = 1.32; 95%CI 1.06, 1.65). Compared with Children living in renting house, children living in purchased house were more likely to receive UTD immunization (OR = 1.61, 95%CI 1.14, 2.27). Compared with children born in hospital at or above the county level, children born at home were less likely to receive UTD immunization (OR = 0.33; 95%CI 0.20, 0.55), but children born in health clinics of towns or in village clinics were same likely to receive UTD immunization.

**Table 3:** Logistic regression analysis of UTD 1:3:3:3:1 series immunization by socio demographic variables, knowledge about vaccine and disease, and attitude toward immunization safety: Guangdong, China, 2011

	**n**	**OR (95% CI)**	**P Value**
Primary caregiver			
Mother	1111	1.00	
Father	257	0.84 (0.63–1.11)	0.219
Other people	162	1.20 (0.84–1.71)	0.321
Primary caregiver’s age (year)			
<25	176	1.00	
25–34	950	0.93 (0.67–1.31)	0.694
>34	404	1.09 (0.75–1.59)	0.641
Primary caregiver’s gender			
Male	302	1.00	
Female	1228	1.28 (0.99–1.66)	0.060
Primary caregiver’s occupation			
Worker	161	1.00	
Commercial staff	313	1.87 (1.27–2.76)	0.002
Professional	54	2.00 (1.04–3.83)	0.038
Private owner	240	2.23 (1.47–3.37)	0.000
Housewife	762	1.67 (1.19–2.35)	0.003
Primary caregiver’s education level			
Primary school and below	539	0.87 (0.63–1.19)	0.373
Middle school	738	1.04 (0.77–1.41)	0.790
High school and above	253	1.00	
Primary caregiver’s Knowledge about vaccine and disease (scores)			
<9	627	1.00	
10–19	647	2.31 (1.83–2.91)	0.000
20–34	256	3.33 (2.38–4.67)	0.000
Primary caregiver’s attitude toward immunization safety (scores)			
0–7	454	1.00	
8–13	1076	1.75 (1.40–2.19)	0.000
Family income (Yuan/person/year)			
<3000	671	1.00	
3000–5000	544	1.27 (1.00–1.60)	0.049
>5000	315	1.50 (1.12–1.99)	0.006
The child’s age (months)			
12–35	1180	1.05 (0.82–1.35)	0.687
36–59	350	1.00	
The child’s sex			
Female	860	0.76 (0.62–0.93)	0.007
Male	670	1.00	
The child’s Hukou			
Guangdong	562	1.32 (1.06–1.65)	0.014
Out of Guangdong	968	1.00	
Residential buildings			
Purchased house	187	1.61 (1.14–2.27)	0.007
Renting house	1343	1.00	
The child’s birth place			0.000
Hospital at or above the county level	780	1.00	
Health clinics of towns	644	0.88 (0.70–1.09)	0.243
Village clinics	40	0.53 (0.28–1.00)	0.050
Home	66	0.33 (0.20–0.55)	0.000

## Discussion

According to our survey, the 5 vaccines’ UTD immunization coverage rates (71.5%–77.6%) of migrant children were lower than the reported immunization coverage rates (higher than 98%) which were reported through the National Immunization Information Surveillance System (NIISS) in recent years and the MOH’s goal of 90% coverage. But the rates were comparable with the previous research in Beijing [17] and other provinces of similar socioeconomic development in China [6,15,18]. And low coverage rates in immigrant children were also reported in other developing and developed countries [19-21]. Low age-appropriate immunization coverage rate was seldom discussed and therefore attracted fewer attention of policy makers. The markedly low immunization coverage rates may affect herd immunity, and they would greatly increase those children’s susceptibility to vaccine preventable diseases. The outbreak of epidemics could become a real threat. Because migrant children accounted for most children in the surveyed areas, the overall immunization coverage of children in these areas still needed to be improved. According to China’s sixth Population Census in 2010 [1], both the amount of Guangdong residential people and migrant people were largest in China and the problem was especially severe in Guangdong.

The immunization coverage for HepB was only 72%. And about 13% of the surveyed children did not receive HepB within 1 day after birth. These results were alarming because Guangdong province was a hepatitis B virus (HBV) high endemic area in China with 13.55% of the hepatitis B surface antigen (HBsAg) positive rate in 2006, the second highest in the country [14]. Only 58.8% of the surveyed children completed the first series of OPV at appropriate age and 16.9% were delayed to accept the 1^st^ dose. There were four wild poliovirus epidemic countries throughout the world in 2011 and three of them were China’s neighboring countries. Wild poliovirus re-entry occurred in Xinjiang province of China in 2011. Re-entry of the wild virus from neighboring countries was possible and could cause outbreaks among susceptible children due to not receiving age-appropriate immunization because of the frequent foreign trade in Guangdong. The coverage of MCV1 was 71.8% but the age-appropriate rate was just 37.2%. There was still a large gap from the goal of 90%. The lower timely coverage rate of MCV1 was considered as the key reason for measles prevalence in the younger age-group of 8 to 12 months. So great efforts were still needed to improve the age-appropriate immunization rates for HepB, OPV and MCV.

The primary caregiver’s determinants associated with UTD immunization rate for the 1:3:3:3:1 series were primary caregiver’s occupation, knowledge about immunization, and attitude toward immunization safety. The child’s determinants were sex, Hukou, birth place and residential buildings. And family income was also an influence factor. There were some specific reasons for them. The primary caregiver’s occupation was significantly associated with the child’s immunization status. Migrant workers in China were mostly manufacture employers and they had little salary. Comparing with other occupational people, workers must go to work on time and the work hour per day was mostly more than 8 hours. Most workers could not spare time to have children vaccinated timely and could not be involved in appropriate health promotion activities aiming at improving child’s vaccination coverage. The primary caregiver’s knowledge about vaccine and disease was the strongest predictor of the child’s immunization status. There was a dose-response relation between them. The primary caregiver’s attitude toward immunization safety contributed to high UTD immunization rate, which was consistent with previous researchers in some developing countries. In China, some adverse propaganda about immunization in recent years may influence people’s immunization awareness. Low confidence about vaccination deterred caregivers from having their children vaccinated. Boys showed higher UTD immunization rate than girls. It indicated son preference toward immunization services in migrant children in China. There was gender discrimination in vaccinations. Children whose Hukou were out of Guangdong tended to receive not UTD immunization and the reason might be that most of them were from undeveloped areas of China. Immunization delivery in these areas still needed to been improved. This might also be partially caused by record scattering or record missing during the frequent migration. Based on incomplete immunization documentation, vaccines could not be administered at appropriate age or intervals by providers [22,23]. For immunization record scattering or record missing, electronic immunization information system would be helpful to facilitate record sharing between clinics and help providers to avoid vaccine spacing errors. Furthermore, the electronic system was important to keep track of migrant children and indicate their migrating trend and it would allow rapid targeting of additional activities. Migrants lived in purchasing buildings were as the registered people. These buildings provided good living conditions and had municipal sanitary system. Their floating was not so frequent and they were easy to be noticed for immunized. Whether the child’s birth took place in hospital or at home was also an influence factor, as has been found in previous studies [24]. This had implications for immunization opportunities, since the BCG and the first dose of HepB were given at birth. In China, mothers who gave birth in hospital usually had more schooling and higher family income. The higher level of hospital where they were in indicated their better economic condition. They might also have more sources of information and more acceptance of medical care in general and preventive health care in particular. Consistent with other studies, lower income was found to be a barrier to child immunization [25,26]. In China migrant children in low income families had been found to be at a disadvantage when it came to access to health service [27]. Most of them lived in poor remote areas and were hardest to reach by the health services and parents might also encounter more barriers in contacting them. Vaccines might arrive at the stations only every other month and that supplies might run out quickly.

It was noteworthy that schooling was not associated with UTD in the five-vaccine series, as has been found in previous researches [28]. This might be due to the homogeneity of the population: 83.5% of the mothers in our study had little schooling.

Our study also had some limitations. First, when written immunization history was missing during migration, the child would be regarded as unvaccinated. So the UTD and age-appropriate immunization coverage rates would be underestimated. However, children without written vaccination history were less likely to being UTD or being age-appropriate [27,29,30]. And only 49 (3.2%) children didn’t have written vaccination history. So the underestimation was slight. The underestimation was inevitable without sharing immunization record between clinics, so the significance of electronic immunization system was necessary. Secondly, there may be a selection bias, because we selected the youngest one when there were two or more eligible children in the surveyed household. It might overestimate the immunization coverage. Thirdly, our results were based on the data from the 29 densely populated towns and townships of migrant children, so the results could not be extended to other areas in Guangdong. But the results would provide important insights for improving immunization coverage of migrant children especially in other towns and townships in suburbs of Guangdong, which had large number of migrants at low socioeconomic level.

## Conclusion

Our findings demonstrated the importance of timely immunization and UTD immunization coverage of the 5-vaccine primary doses among migrant children in Guangdong and they should be closely monitored and assessed by the NIISS. The results would provide more important insights for future policy of public health. First, increasing financial and labor input to immunization clinics should be primarily emphasized by policymakers. Input should be sufficient to sustain the work load of vaccinating not only registered children but also more and more migrant children. Second, strategies to improve age-appropriate immunization coverage rate in Guangdong province could be explored through both immunization user and provider. Except for “passive” clinical services, active out-reach activities including notification services and supplementary immunization activities (SIAs) should be more emphasized. Healthcare workers should make house-to-house visit to identify incompletely vaccinated children and immunized them in clinics. Third, Electronic immunization information system should be used to facilitate record sharing between clinics, keep track of migrant children and avoid vaccine spacing errors. At last, health education should been emphasized so as to encourage caregivers to follow the immunization program for their children. Hospital delivery also should be advocated to increase the immunization opportunities.

## Abbreviations

BCG: Bacille calmette guerin; DTP: Diphtheria, tetanus and pertussis combined vaccine; HepB: Hepatitis B vaccine; OPV: Oral poliomyelitis vaccine; MCV: Measles-containing vaccine; EPI: Expanded program of immunization; UNICEF: United Nations International Children’s Emergency Fund; UTD: Up-to-date; MOH: Ministry of health; OR: Odds ratio; NIISS: National Immunization Information Surveillance System; HBV: Hepatitis B virus; HBsAg: Hepatitis B surface antigen; SIAs: Supplementary immunization activities; WHO: World health organization; PPS: Probability-proportionate-to-size; CDC: Center for disease control and prevention; AEFI: Adverse events following immunization

## Competing interests

The authors declare that they have no competing interests.

## Authors’ contributions

HZ (Principal Investigator) had the original idea for the study and led the design of the study and application for grant funding. KH designed the study, collected the data, analyzed, interpreted the data, handled supervision, and prepared the manuscript. ZH, HZ, QQ and BC collected the data, analyzed the data, interpreted the data and handled supervision. JX interpreted the data and made critical revisions to the manuscript. All authors read and approved the final manuscript.

## Pre-publication history

The pre-publication history for this paper can be accessed here:

http://www.biomedcentral.com/1471-2458/14/203/prepub

## Supplementary Material

Additional file 1: Questionnaire content of immunization knowledge, attitude towards immunization safety and children written immunization history: Guangdong, China, 2011. Click here for file
