# Supplementary material for: Vaccination coverage and its determinants among migrant children in Guangdong, China
Source: BMC Public Health. 2014 Feb 26;14:203. doi: 10.1186/1471-2458-14-203 (PMC3938078; doi:10.1186/1471-2458-14-203)
Supplement: Additional file 1: — Questionnaire content of immunization knowledge, attitude towards immunization safety and children written immunization history: Guangdong, China, 2011. [file 1471-2458-14-203-S1.doc]

**File 1 Questionnaire content of immunization knowledge, attitude towards immunization safety and children written immunization history: Guangdong, China, 2011**

**一、Immunization Knowledge**

1.Should children be immunized by vaccines after birth?

1=Yes 2=No 3=Not clear

1. Should children with cough or fever be immunized by vaccines?

1=Yes 2=No 3=Not clear

3.Should children be observed about their response after vaccination?

1=Yes 2=No 3=Not clear

1. How many minutes should children be observed after vaccination? minutes.
2. What vaccines are provided for free by our government?

1=Hepatitis B Vaccine 2=BCG Vaccine 3=Poliomyelitis Vaccine 4=DPT Vaccine

5=DT Vaccine 6=Measles Vaccine 7=Encephalitis Vaccine 8=Epidemic Menigitis

Vaccine for type A 9=Epidemic Menigitis Vaccine for Type A and C 10=Measles and

Rubella Vaccine 11=Measles and Mumps Vaccine 12=Measles, Rubella and Mumps

Vaccine 13=Hepatitis A Vaccine 14=Influenza Vaccine 15=Varicella Vaccine

16=Pneumonia Vaccine

6.What diseases can be prevented by the vaccines?

1=Hepatitis B 2=Tuberculosis 3=Poliomyelitis 4=Pertussis 5=Diphtheria 6=Tetanus

7=Measles 8=Encephalitis 9=Epidemic Menigitis 10=Rubella 11=Mumps

12=Hepatitis A 13=Influenza 14=Varicella Vaccine 15=Pneumonia

**二、Attitude towards immunization safety**

1. Do you think vaccines are safe?

1=All are safe 2=Most of them are safe 3=Some of them are safe

4=Few of them are safe 5=None are safe

1. Do you think vaccines are helpful to your child’s health?

1=All are helpful 2=Most of them are helpful 3=Some of them are helpful

4=Few of them are helpful 5=None are helpful

9.Do you think the adverse events following immunization will occur?

1=Each time 2=Most of time 3=Sometimes 4=Seldom 5=Never

**三、Children written immunization history**

| Vaccine | Dose | Immunization time |
| --- | --- | --- |
| BCG vaccine |  | Year Month Day |
| Poliomyelitis Vaccine | 1 | Year Month Day |
|  | 2 | Year Month Day |
|  | 3 | Year Month Day |
|  | 4 | Year Month Day |
| DPT Vaccine | 1 | Year Month Day |
|  | 2 | Year Month Day |
|  | 3 | Year Month Day |
|  | 4 | Year Month Day |
| Measles-containing Vaccine | 1 | Year Month Day |
|  | 2 | Year Month Day |
| Hepatitis B Vaccine | 1 | Year Month Day |
|  | 2 | Year Month Day |
|  | 3 | Year Month Day |
